# Supplementary material for: A Radiomic-Based Machine Learning Model Predicts Endometrial Cancer Recurrence Using Preoperative CT Radiomic Features: A Pilot Study
Source: Cancers (Basel). 2023 Sep 13;15(18):4534. doi: 10.3390/cancers15184534 (PMC10526953; doi:10.3390/cancers15184534)
Supplement: Supplementary file 1 [file cancers-15-04534-s001.zip › cancers-2548932-supplementary.pdf]

Supplementary methods

Surrogate TCGA Molecular/PROMISE/PORTEC analysis

POLE mutational analysis was done by means of Next Generation Sequencing (NGS) analysis on an Ion torrent platform as previously described. Immunohistochemistry (IHC) was used to evaluate the expression of p53, MLH1, PMS2, MSH2, MSH6 as previously described [1,2]. The cases were considered mismatch repair deficient (MMRd) if one of the four proteins was absent or if MLH1/PMS2 or MSH2/MSH6 were negative. In case of tumors with loss of MLH1 protein expression, further testing for the methylation status of the 5′ regulatory region of MLH1 was performed as previously described. In case of mismatch repair (MMR) proteins (MLH1, PMS2, MSH2, and MSH6) expression, negative scores were assigned if no nuclear immunostaining was present. EC tumors were classified according to the latest TCGA molecular group [3]. The POLE "ultramutated" group was assigned after the diagnostic interpretation of *POLE* mutations according to reported guidelines [4]. If MMR deficiency (MMRd) was found and no *POLE* mutations were present, the MMRd "hypermutated" group was assigned. Subsequently, if p53 expression pattern was abnormal, the p53abn "copy number-high/serous-like" group was assigned. In case of tumors without any POLE mutations, normal MMR and p53 expression, the NSMP "No specific molecular profile" corresponding to the "copy number-low" TCGA subgroup was assigned.

1. Singh, N.; Piskorz, A.M.; Bosse, T.; Jimenez-Linan, M.; Rous, B.; Brenton, J.D.; Gilks, C.B.; Köbel, M. P53 Immunohistochemistry Is an Accurate Surrogate for TP53 Mutational Analysis in Endometrial Carcinoma Biopsies. *J. Pathol.* **2020**, *250*, 336–345, doi:10.1002/path.5375.
2. Dondi, G.; Coluccelli, S.; De Leo, A.; Ferrari, S.; Gruppioni, E.; Bovicelli, A.; Godino, L.; Coadă, C.A.; Morganti, A.G.; Giordano, A.; et al. An Analysis of Clinical, Surgical, Pathological and Molecular Characteristics of Endometrial Cancer According to Mismatch Repair Status. A Multidisciplinary Approach. *Int. J. Mol. Sci.* **2020**, *21*, E7188, doi:10.3390/ijms21197188.
3. Levine, D.A. Integrated Genomic Characterization of Endometrial Carcinoma. *Nature* **2013**, *497*, 67–73, doi:10.1038/nature12113.
4. León-Castillo, A.; Britton, H.; McConechy, M.K.; McAlpine, J.N.; Nout, R.; Kommoss, S.; Brucker, S.Y.; Carlson, J.W.; Epstein, E.; Rau, T.T.; et al. Interpretation of Somatic POLE Mutations in Endometrial Carcinoma. *J. Pathol.* **2020**, *250*, 323–335, doi:10.1002/path.5372.

Supplementary results

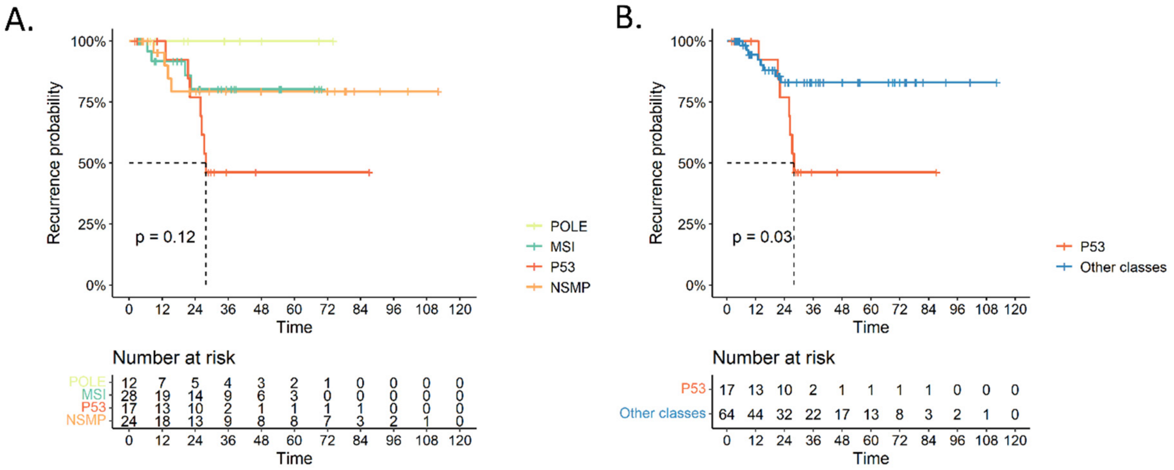

Supplementary Figure S1. Kaplan-Meier curves for PFS of EC patients based on the TCGA/PROMISE classification (A) and when considering only the P53 mutated class vs all other classes (B).
